# Two-level mixed-effects height to crown base model for moso bamboo (*Phyllostachys edulis*) in Eastern China

**DOI:** 10.3389/fpls.2023.1095126

**Published:** 2023-03-30

**Authors:** Xiao Zhou, Yang Zhou, Xuan Zhang, Ram P. Sharma, Fengying Guan, Shaohui Fan, Guanglu Liu

**Affiliations:** ^1^ International Center for Bamboo and Rattan, Key Laboratory of National Forestry and Grassland Administration, Beijing, China; ^2^ National Location Observation and Research Station of the Bamboo Forest Ecosystem in Yixing, National Forestry and Grassland Administration, Yixing, China; ^3^ Institute of Forestry, Tribhuwan University, Kritipur, Kathmandu, Nepal

**Keywords:** random effects prediction, mean response, crown density, BAL, sample selection strategy, response calibration, logistic function

## Abstract

Height to crown base (HCB) is an important predictor variable for forest growth and yield models and is of great significance for bamboo stem utilization. However, existing HCB models built so far on the hierarchically structured data are for arbor forests, and not applied to bamboo forests. Based on the fitting of data acquired from 38 temporary sample plots of *Phyllostachys edulis* forests in Yixing, Jiangsu Province, we selected the best HCB model (logistic model) from among six basic models and extended it by integrating predictor variables, which involved evaluating the impact of 13 variables on HCB. Block- and sample plot-level random effects were introduced to the extended model to account for nested data structures through mixed-effects modeling. The results showed that bamboo height, diameter at breast height, total basal area of all bamboo individuals with a diameter larger than that of the subject bamboo, and canopy density contributed significantly more to variation in HCB than other variables did. Introducing two-level random effects resulted in a significant improvement in the accuracy of the model. Different sampling strategies were evaluated for response calibration (model localization), and the optimal strategy was identified. The prediction accuracy of the HCB model was substantially improved, with an increase in the number of bamboo samples in the calibration. Based on our findings, we recommend the use of four randomly selected bamboo individuals per sample to provide a compromise between measurement cost, model use efficiency, and prediction accuracy.

## 1 Introduction

According to the 2022 Sixth Assessment Report by the Intergovernmental Panel on Climate Change (IPCC), the concentration of CO_2_ in the atmosphere is now 410 ppm, which is the highest level in recent years. Afforestation can mitigate climate change by reducing atmospheric CO_2_ (Wang et al., 2013). Although the planet’s arboreal forest area is decreasing dramatically worldwide, the area of bamboo forests is increasing substantially (FAO, 2010). Bamboo forests have distinct characteristics from arboreal forests, including faster growth, higher production efficiency, and faster maturity. Bamboo forests has steadily increased their net carbon storage capacity in recent decades, becoming a carbon sink in the subtropical region of China (Chen et al., 2009; Wang et al., 2013) and playing an important role in mitigating global warming (Yen and Lee, 2011; Yen, 2015; Yen, 2016; Zhou et al., 2022).

The bamboo crown is an important part of the bamboo forest, as it is where photosynthesis, transpiration, and other physiological processes occur (Morataya et al., 1999). Bamboo crowns affect the distribution of the biomass of various organs in bamboo forests, the density and dry matter accumulation of bamboo stems, and the quality of bamboo stems (Biging and Dobbertin, 1992; Chmura et al., 2007). Bamboo crowns also reflect the vitality of bamboo forests (Assman, 1970; Hasenauer and Monserud, 1996; Pan et al., 2020), the quality of stems (Kuprevicius et al., 2014; Yang, 2017), and wind resistance (Ancelin et al., 2004; Yang, 2017).

The ratio of crown length to bamboo height (CR) is an important parameter for quantifying and determining bamboo vitality, competition, stability of growth stage, and production efficiency (Monserud and Sterba, 1996; Brown et al., 2004; Kuprevicius et al., 2014). Bamboo CR can be measured directly (Maguire and Hann, 1990) or determined by measuring total bamboo height (H) and height to crown base (HCB) (Fu et al., 2017; Sharma et al., 2017; Pan et al., 2020; Yang et al., 2020). HCB is defined as the vertical height from the ground to the first living branch (Hasenauer and Monserud, 1996). HCB reflects the utilization efficiency of bamboo stems; that is, the smaller the distance between bamboo branches and the ground, the lower the availability of bamboo stems (Zhou, 1998; Sun et al., 2009; Li et al., 2010; Sun, 2010). The stem is the most-utilized part of bamboo and can be used as an industrial raw material to produce paper, chopsticks, charcoal, etc. (Zhou, 1998; Dam et al., 2018). Therefore, the study of bamboo HCB is of great practical significance for nutrition research (photosynthesis and transpiration of the bamboo canopy) and utilization research (bamboo stem quality). However, while conducting bamboo forest surveys or measurements, especially in dense bamboo forests, it is difficult to distinguish bamboo crowns; thus, HCB measurement is time-consuming, laborious, and costly (Temesgen et al., 2005; Zhou et al., 2022). To avoid these difficulties, a model to estimate HCB should be developed using measured data from a sufficient number of bamboo plants.

HCB models for different tree species have been established, and their construction methods vary from simple to complex (Wykoff et al., 1982; Van Deusen and Biging, 1985; Walters and Hann, 1986; Popoola and Adesoye, 2012; Rijal et al., 2012; Fu et al., 2017; Sharma et al., 2017; Yang et al., 2020; Zhou et al., 2022). The predictor variables used in these models include individual tree variables (H, diameter at breast height; DBH; and competition-related variables) and stand variables (stand density, base area, and site quality-describing variables). The general forms of existing HCB models are exponential and logistic (Fu et al., 2017; Sharma et al., 2017; Yang et al., 2020). Most existing HCB models use ordinary least squares (OLS) regression for parameter estimation, and the predictor variables included in the models are simple. Bamboo forest growth differs significantly by block and sample plot nested in the block, and repeated measurements of different bamboo attributes might be made during the inventory. Using OLS to estimate HCB models built on a hierarchical (nested) data structure would lead to biased parameter estimation and invalid hypothesis tests (West et al., 1984). Mixed-effects modeling for such a nested data structure (e.g., bamboo culms within a sample plot, sample plots within a block, multiple measurements on the same plot or bamboo plant) provides the most robust method to avoid the problems caused by a nested data structure, including problems with spatial correlations. Mixed-effects modeling has been widely used in forest modeling in recent years because of its high efficiency and robustness (Fu et al., 2017; Sharma et al., 2017; Pan et al., 2020; Yang et al., 2020; Zhou et al., 2021a; Zhou et al., 2021b). Only a few HCB modeling studies exist, and they are based solely on arboreal species (Fu et al., 2017; Pan et al., 2020; Yang et al., 2020) and use one-level mixed-effects modeling. Here, we use two-level mixed-effects modeling, which is novel in the field of bamboo forest modeling. Considering the significant contribution of bamboo forests to mitigating global warming and their importance in balancing ecosystem function, building a bamboo HCB model would be worthwhile.

To solve the problems (nested data structure, spatial correlations, lack of intensive bamboo HCB research, etc.), this study developed a mixed-effects HCB model by integrating DBH, total basal area of all bamboo individuals with a diameter larger than that of the subject bamboo (BAL), and canopy density (CD) as the predictor variables and random effects at the block and sample plot levels. Specifically, this study aimed to (1) develop a nonlinear mixed-effects (NLME) model of bamboo HCB, (2) quantify the impact of the predictor variables on HCB, and (3) select the strategies that would be most suitable for a response calibration of the two-level NLME HCB model. We used temporary sample plot data of *Phyllostachys edulis* (moso bamboo) forests on the Yixing Forestry Farm, Jiangsu Province, for our study. The methods and results presented can provide an important reference for efficient inventorying and effective management of bamboo forests.

## 2 Materials and methods

### 2.1 Study area and data

**Figure 1:**
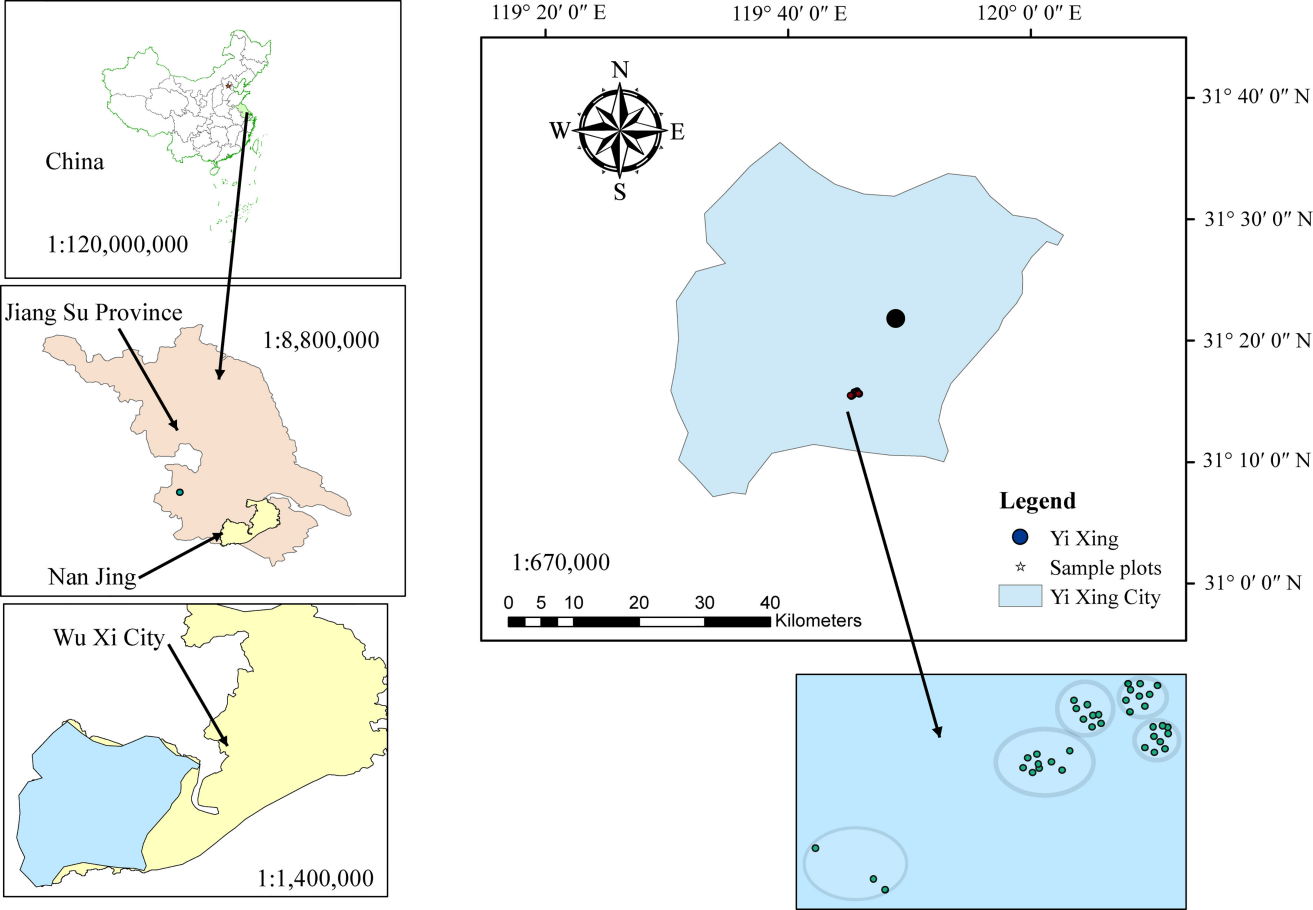
Location of sample plots nested in different block settings.

The study area is located in the Yixing State-owned Forest Farm, Jiangsu, China (119° 41 ′ 38 ″ E, 31° 13 ′12 ″N) (**Figure 1**). The forest farm is humid throughout the year and has a subtropical monsoonal climate. It has an annual average temperature of 16.7 °C, total precipitation of 1805.4 mm, 1807.5 h of sunshine, and 150 rainy days. It is the area with the richest bamboo forest resources in Jiangsu Province.

**Figure 2:**
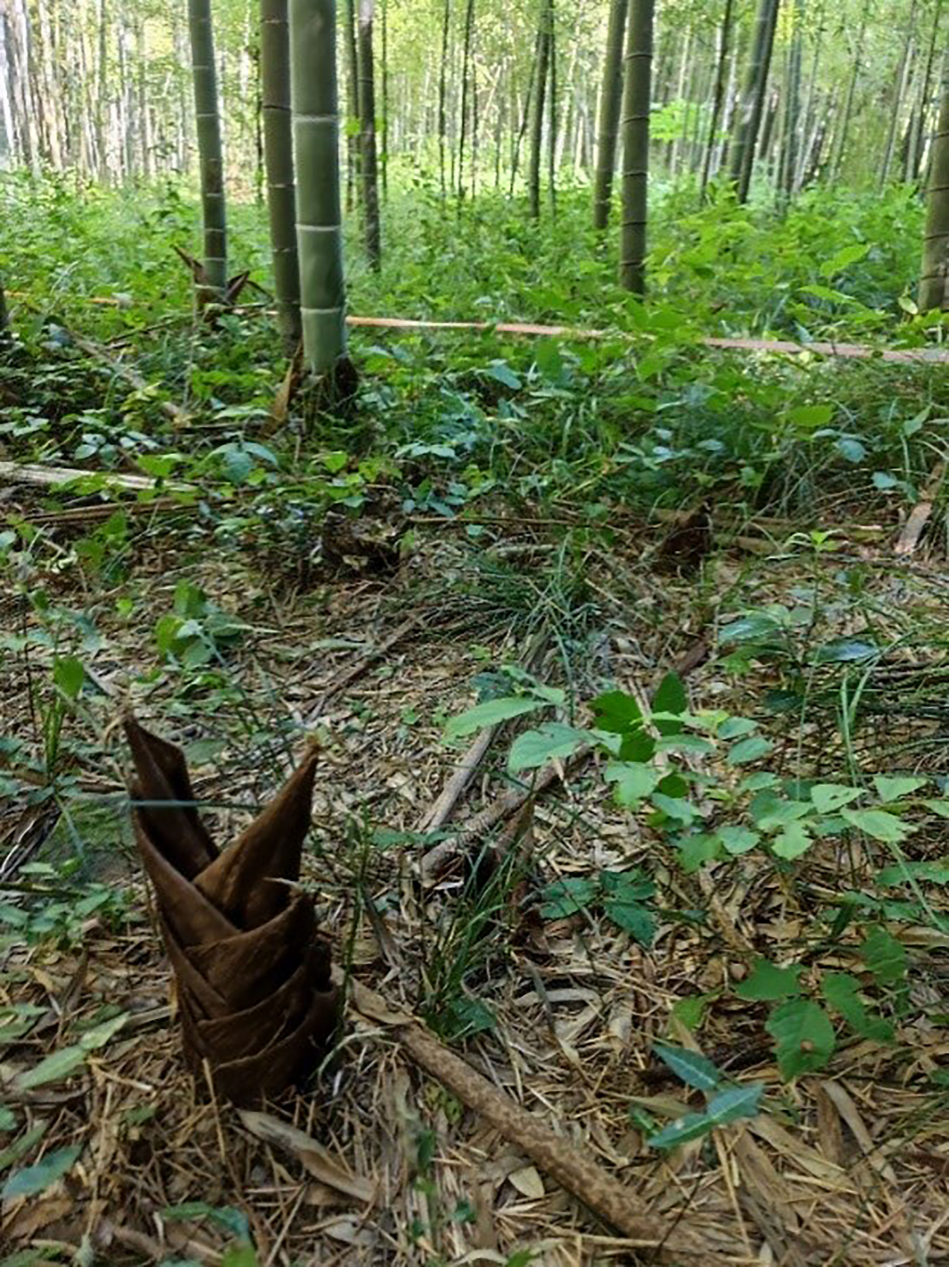
Features of moso bamboo in the sample plot.

**Figure 3:**
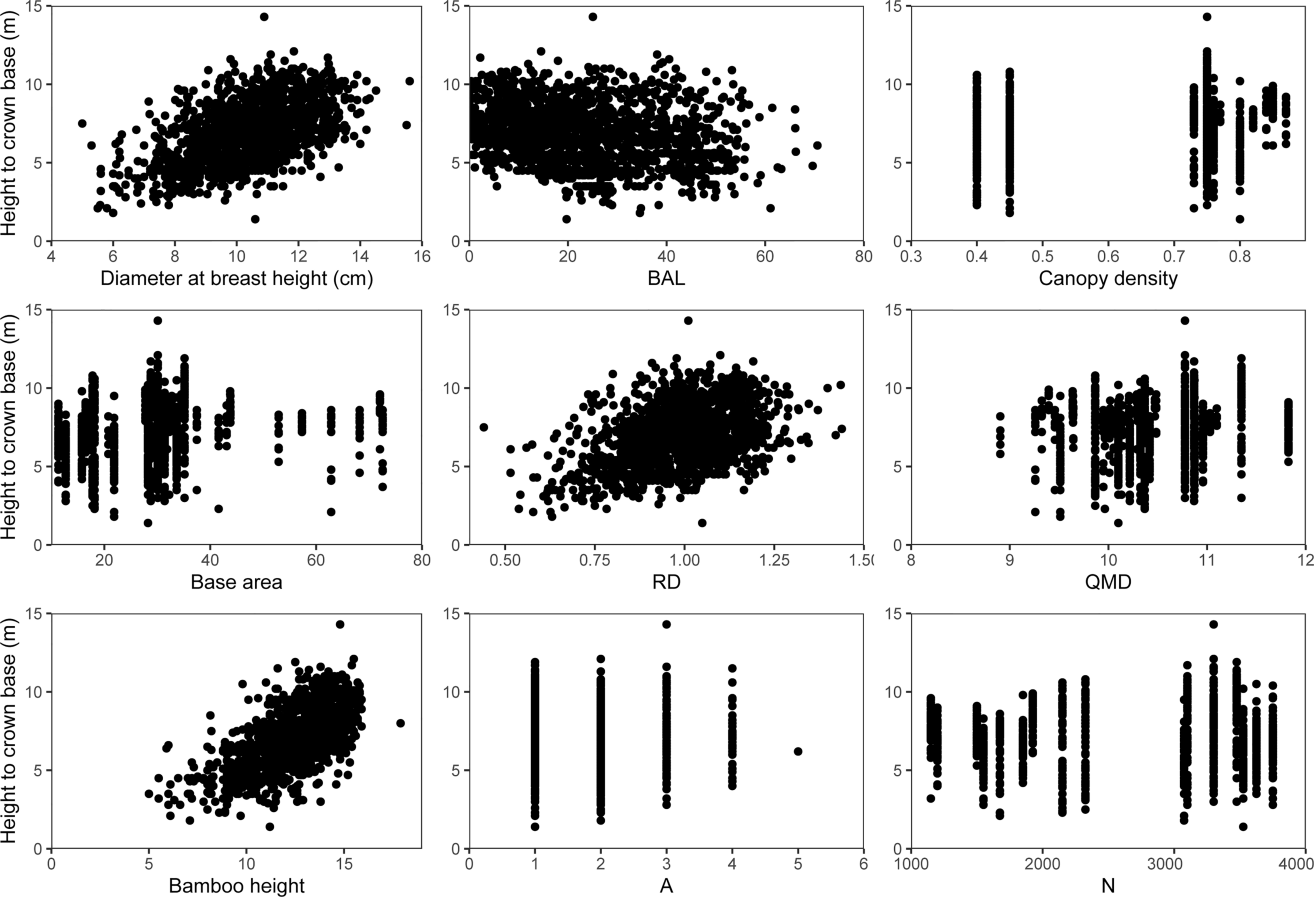
Scatter plots of HCB versus different predictor variables evaluated for their potential contribution to the HCB model of moso bamboo.

From July to August 2022, 38 temporary sample plots (**Figure 1**) were established on the forest farm and investigated. The area of each sample plot was 20 × 20 m. These sample plots represented most of the forest structure, forest size, and vitality in the region. The sample plots were distributed across five different blocks. Each bamboo plant in the sample plots with a DBH greater than 5 cm was measured for DBH, H, height to crown base (HCB), crown width, canopy density, and age. A total of 1374 individuals were measured and the measured stand and individual bamboo data are summarized in **Table 1**. Because of the unique growth characteristics of moso bamboo, with a vegetative cycle of two years (on-year and off-year), the age could be expressed as “du” (Tang et al., 2016), with one (I) “du” corresponding to 1–2 years, and 2 (II) and 3 (III) “du” corresponding to 3–4 and 5–6 years, respectively (Tang et al., 2016). **Figure 2** shows the general features of bamboo in the study area. **Figure 3** shows the scatter plots of HCB versus potential predictor variables evaluated in our study.

**Table 1:** Bamboo forest variables statistics.

Variable	Min	Max	Mean	SD
DBH (cm)	5.0	15.6	10.38	1.57
QMD (cm)	8.91	11.8	10.47	0.60
RD	0.44	1.44	0.9913	0.12
HCB (m)	1.4	14.3	6.88	1.87
H (m)	5	17.9	12.16	2.01
BAL (m^2^ ha^−1^)	0	70.58	23.44	14.62
A(du)	1	5	1.67	0.78
CW(m)	2.03	4.86	3.09	0.38
BA (m^2^ ha^−1^)	11.32	72.56	28.2	11.23
CD	0.4	0.87	0.65	0.16
N (culms ha^−1^)	1150	3750	2783	853

Diameter at breast height (DBH), quadratic mean DBH (QMD), relative diameter (ratio of DBH of an individual to QMD; RD), height to crown base (HCB), bamboo height (H), total basal area of all bamboos with a diameter larger than that of the subject bamboo (BAL), bamboo age (A), crown width (CW), base area per hectare (BA), canopy density (CD), number of culms per hectare (stand density; N), minimum (Min), maximum (Max), the average value (Mean), and standard deviation (SD).

### 2.2 Basic model

By consulting the relevant literature on HCB modeling, we used six common mathematical functions as candidates for bamboo forest modeling (**Table 2**). As we found that the DBH and BAL of bamboo were significantly related to HCB, which was also proven by a recent bamboo modeling study (Zhou et al., 2022), we used these variables as predictor variables in the basic models. We then used the entire data set to fit these models, and four versatile evaluation indicators (Eqs. 1–4) were used to compare model fits. The model with the best-fitting effect was selected for further extension and analysis.

**Table 2:** HCB candidate models considered (HCB: height to crown base; H: bamboo height; x: vector of bamboo variables; β, a, c, 1/m and W: parameter vector; ∞ : infinity).

Designation	Mathematical Form	Name of Function	Value Range	Source
M1	HCB=H[1−exp(βx)]	Exponential	(−∞,H)	Wykoff et al., 1982
M2	$HCB=H[1-\text{exp(}\beta x{)}^{2}]$	Exponential	(−∞,H)	Van Deusen and Biging, 1985
M3	$HCB=\frac{H}{\left[1+\exp(\beta x)\right]}$	Logistic	(0,H)	Walters and Hann, 1986
M4	$HCB=\frac{H}{{\left[1+c\exp(\beta x)\right]}^{1/m}}$	Logistic	(0,H)	Rijal et al. (2012)
M5	$HCB=H[1-c\text{exp}(\beta x^{W})]$	Exponential	(−∞,H)	Rijal et al. (2012)
M6	HCB=H[a+exp(βx)]	Exponential	(−∞,H)	Popoola and Adesoye (2012)


(1) $$MD=\frac{1}{n}\sum_{i=1}^{n}\left(HCB_{ijk}-{\hat{HCB}}_{ijk}\right)$$

(2) $$R^{2}=1-\sum_{i=1}^{n}{\left(HCB_{ijk}-{\hat{HCB}}_{ijk}\right)}^{2}/\sum_{i=1}^{n}{\left(HCB_{ijk}-\frac{\sum_{i=1}^{n}HCB_{ijk}}{n}\right)}^{2}$$

(3) $$RMSE=\sqrt{\frac{1}{n}\sum_{i=1}^{n}{\left(HCB_{ijk}-{\hat{HCB}}_{ijk}\right)}^{2}}$$

(4) $$TRE=\sum_{i=1}^{n}\left|HCB_{ijk}-{\hat{HCB}}_{ijk}\right|/\sum_{i=1}^{n}{\hat{HCB}}_{ijk}$$

where $HCB_{ijk}$ is the height to crown base of the k^th^ bamboo in the j^th^ sample plot in the i^th^ block, ${\hat{HCB}}_{ijk}$ is the estimated value of the k^th^ bamboo in the j^th^ sample plot in the i^th^ block, and n is the number of sample plots.

In addition to DBH, BAL, and bamboo size, location and competition also affect HCB (Fu et al., 2017; Pan et al., 2020; Yang et al., 2020). We therefore also considered bamboo size, stand vitality, site quality, and competition. There were 12 predictor variables in total, which can be grouped into the bamboo size and stand vitality variables (H, CW, CD, bamboo age (A), stand density (N), base area (BA), quadratic mean DBH; QMD), competition-related variables (total basal area of all bamboo individuals with a diameter larger than that of the subject bamboo (BAL) and relative diameter (RD; the ratio of DBH of individual bamboo to QMD), and site quality factors (slope degree, slope position, and humus thickness), and their impact on HCB was evaluated.

We used graphical analysis and appropriate statistical tests to select the variables that contributed the most to the HCB models (Uzoh and Oliver, 2008). Root mean square error (RMSE) and Akaike’s information criterion (AIC) were used to compare the model variants created by different combinations and logarithmic transformations of the predictor variables. All analyses were carried out using the R *nls* function, and the model with the most attractive fit statistics was selected for further extension using two-level mixed-effects modeling.

### 2.3 Two-level NLME HCB model

We introduced random effects at both the block and sample plot levels by considering the combination of each fixed parameter and random effects and selecting the best combination using AIC and Log Likelihood (LL). Spatial correlation appeared to have little influence on the HCB model; thus, we disregarded this effect. However, there was a significant heteroscedasticity problem, which was reduced by using a variance-covariance matrix (Davidian and Giltiman, 1995).


(5) $$R_{i}=\sigma^{2}G_{i}^{0.5}\Gamma_{i}G_{i}^{0.5}$$

where $R_{i}$ the variance-covariance matrix, $\sigma^{2}$ the residual variance (Gregoire et al., 1995), $G_{i}$ is the diagonal matrix describing heteroscedasticity and $\Gamma_{i}$ is the matrix describing the spatial correlation. Therefore, $\Gamma_{i}$ is assumed to be an identity matrix.

To reduce the heteroscedasticity problem in Eq. 5, we evaluated three common variance functions (Eqs. 6–8) (Yang et al., 2020; Zhou et al., 2022) and selected the best-performing one. AIC and LL were used to evaluate the effects of the variance functions.


(6) $$Var(\xi_{ijk})=\sigma^{2}\exp(2\gamma H_{ijk})$$

(7) $$Var(\xi_{ijk})=\sigma^{2}H_{ijk}^{2\gamma }$$

(8) $$Var(\xi_{ijk})=\sigma^{2}{\left(\gamma_{1}+H_{ijk}^{2\gamma_{2}}\right)}^{2}$$

where $H_{ijk}$ is the height of the k^th^ bamboo in the j^th^ sample plot in the i^th^ block and $\gamma ,\gamma_{1},\gamma_{2}$ represents the parameter to be estimated.

### 2.4 Parameter estimation

All the basic models (**Table 2**) were estimated using the R *nls* function, and the parameters of the mixed-effects model were estimated using the R *nlme* function (R Core Team, 2020).

### 2.5 Response calibration/prediction with NLME models

Based on the optimal mixed-effects model selected above, we considered two cases: one with only the fixed parameters considered (excluding random effects), termed the M response, and one with local measurements used to estimate random effects, termed model localization or model calibration (Calama and Montero, 2004; Yang et al., 2009). When random effects cannot be estimated, the M response must be used for HCB prediction. The empirical best linear unbiased prediction (EBLUP) theory (Eq. 9) was used to estimate random effects (Lindstrom and Bates, 1990; Liu and Cela, 2008).


$${\hat{u}}_{i}=\hat{\Psi }Z_{i}^{T}{\left({\hat{R}}_{i}+Z_{i}\hat{\Psi }Z_{i}^{T}\right)}^{-1}e_{i}$$

(9) $$=\hat{\Psi }Z_{i}^{T}{\left({\hat{R}}_{i}+Z_{i}\hat{\Psi }Z_{i}^{T}\right)}^{-1}\left[y_{i}-f(\hat{\beta },u_{i}^{*},x_{i})+Z_{i}u_{i}^{*}\right]$$

where ${\hat{u}}_{i}$ is the estimated random effects; $u_{i}^{*}$ is estimated value by using the EPBLU method; f(·) is an NLME HCB model; $\hat{\beta }$ is the fixed effect parameters vector; $x_{i}$ is the vector of predictor variables; $\hat{\Psi }$ is the random effect variance-covariance matrix; ${\hat{R}}_{i}$ is variance-covariance matrix; and $Z_{i}$ is $n_{i}\times q$ dimensional design matrix of the partial derivatives of the NLME HCB model f(·) with respect to the random-effect $u_{i}$ . More detailed descriptions of the response calibration can be found in Meng and Huang (2009) and Fu et al. (2017).

Different sample sizes can be used for response calibration or random-effects estimation. Different sampling strategies produce different random effect values, which affect the prediction accuracy of NLME models. Previous forest modeling studies attempted to identify the optimal sample size for estimating random effects in several forestry NLME models (Meng and Huang, 2009; Yang et al., 2009; Yang et al., 2020; Zhou et al., 2021a). However, none of these studied the NLME HCB model of bamboo forests. As moso bamboo was uniaxially scattered throughout the sample plot, with each bamboo plant growing independently like a tree and many individual plants per sample plot, we were able to evaluate four different sampling strategies for predicting random effects:

1. 1–8 randomly selected bamboo plants per sample plot.
2. 1–8 bamboo plants with the largest DBH per sample plot.
3. 1–8 bamboo plants with average DBH per sample plot.
4. 1–8 bamboo plants with the smallest DBH per sample plot.

RMSE and total relative error (TRE) statistics were used to evaluate the prediction performance of each sampling strategy. The EBLUP theory was used to estimate the empirical Bayesian estimates of random effects. To ensure the stability of the estimates, each strategy was repeated 100 times, and the mean RMSE and TRE statistics were computed for each strategy.

### 2.6 Model evaluation

The effectiveness of the NLME-HCB model could be evaluated by using independent data. However, these data are laborious and costly to gather. Thus, this study adopted the leave-one-out cross-validation (LOOCV) approach (Fu et al., 2018; Yang et al., 2020; Zhou et al., 2021a). We removed one sample plot at a time and fitted the models using data from the remaining 37 sample plots. This process was performed randomly 38 times to ensure that data from each sample plot was excluded from the fitting. The statistical indicators (Eq. 1–4) were computed using the predicted and observed HCB values.

## 3 Results

### 3.1 Basic models

Using all the data, we fitted six candidate models (M1–6; **Table 1**) and evaluated them using four statistical indicators (Eqs. 1–4). All the models had a similar fitting effect. However, the R^2^, RMSE, and TRE of M3, M4, and M5 were slightly higher than those of the other models (**Table 3**). Because the number of parameters of M4 and M5 was higher than those of M3, we chose the simplest model, M3, for further analyses.

**Table 3:** Fit statistics of the basic models.

Model	MB	RMSE	TRE	R^2^
M1	0.0050	1.2970	3.4250	0.5178
M2	0.0050	1.2970	3.4250	0.5178
M3	0.0053	1.2948	3.4129	0.5194
M4	0.0053	1.2948	3.4129	0.5194
M5	0.0052	1.2949	3.3992	0.5194
M6	0.0044	1.2970	3.4244	0.5178

MB, mean bias; RMSE, root mean square error; TRE, total relative error; and R^2^, coefficient of determination.


(10) $$HCB_{ijk}=\frac{H_{ijk}}{\left[1+e^{\left(\beta_{1}+\beta_{2}DBH_{ijk}+\beta_{3}BAL_{ijk}\right)}\right]}+\xi_{ijk}$$

where $HCB_{ijk}$ is the height to crown base of the k^th^ bamboo in the j^th^ sample plot in the i^th^ block, $DBH_{ijk}$ is the DBH of the k^th^ bamboo in the j^th^ sample plot in the i^th^ block, $BAL_{ijk}$ is the BAL of the k^th^ bamboo in the j^th^ sample plot in the i^th^ block, $\beta_{1},\beta_{2},\beta_{3}$ are fixed parameters to be estimated, and $\xi_{ijk}$ is the error term.

### 3.2 Expansion of the basic model

In addition to DBH and BAL, we considered other variables as predictors that substantially affected HCB. The HCB model with DBH, BAL, and CD provided the best fit (R^2^ = 0.5226, RMSE = 1.2905, TRE = 3.3892), indicating that only CD provided a better fitting effect than the other variables evaluated. The use of only three predictor variables in the model prevents over-parameterization and non-convergence problems and still describes the majority of HCB variations. The extended HCB model form is


(11) $$HCB_{ijk}=\frac{H_{ijk}}{\left[1+e^{\left(\beta_{1}+\beta_{2}DBH_{ijk}+\beta_{3}BAL_{ijk}+\beta_{4}CD_{ij}\right)}\right]}+\xi_{ijk}$$

where $HCB_{ijk}$ is the height to crown base of the k^th^ bamboo in the j^th^ sample plot in the i^th^ block, $DBH_{ijk}$ is the DBH of the k^th^ bamboo in the j^th^ sample plot in the i^th^ block, $BAL_{ijk}$ is the BAL of the k^th^ bamboo in the j^th^ sample plot in the i^th^ block and $CD_{ij}$ is the CD of the j^th^ sample plot in the i^th^ block, $\beta_{1},\beta_{2},\beta_{3},\beta_{4}$ are fixed parameters to be estimated, and $\xi_{ijk}$ is the error term.

**Figure 4:**
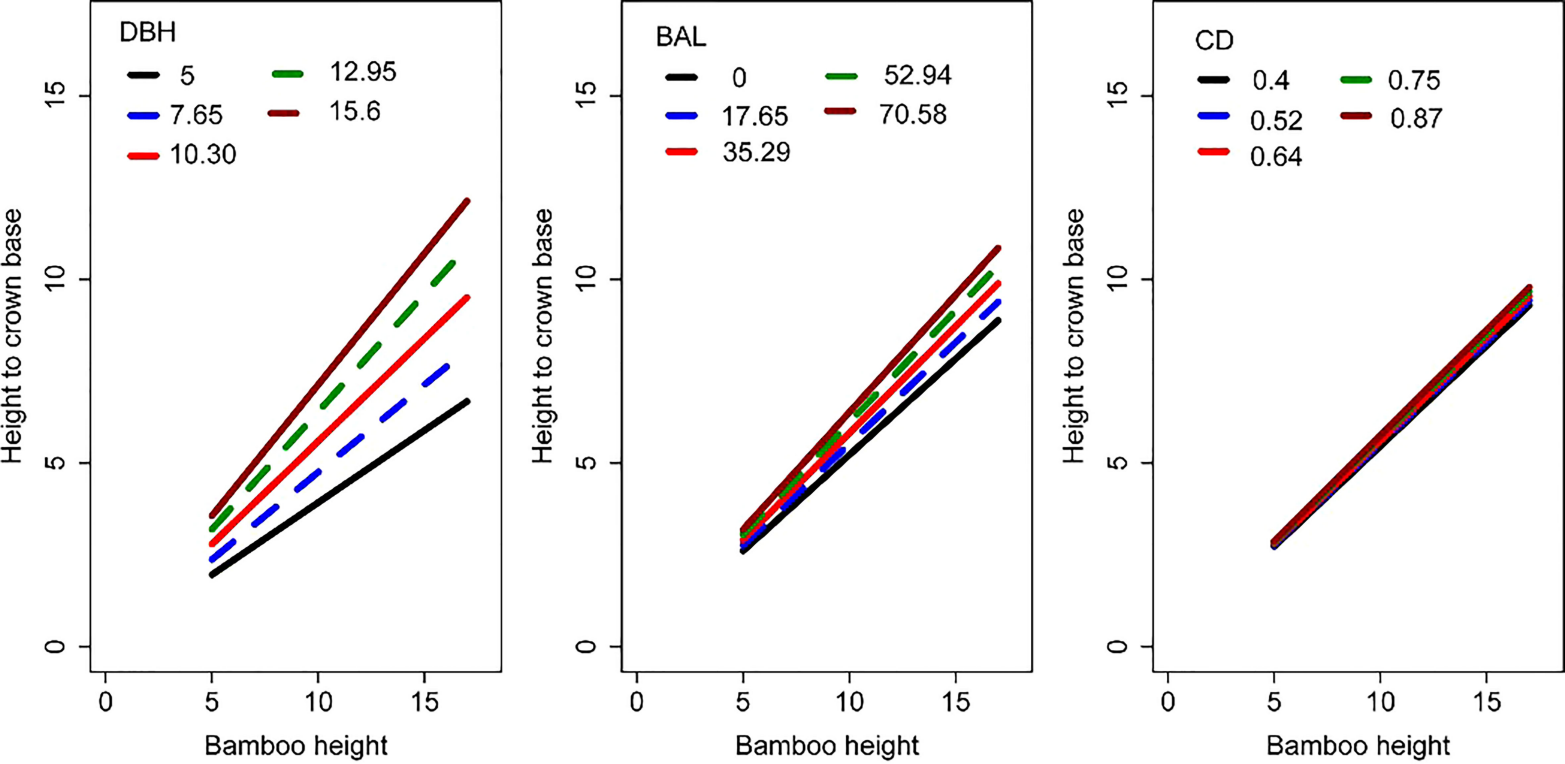
Simulation of the impact of DBH, BAL, and CD on HCB. The HCB model used for this simulation is Eq. 11.

We simulated the effects of DBH, BAL, and CD on HCB estimation (**Figure 4**). HCB increased with increasing BAL, DBH, and CD, with DBH having the greatest impact on HCB, followed by BAL and CD.

**Figure 5:**
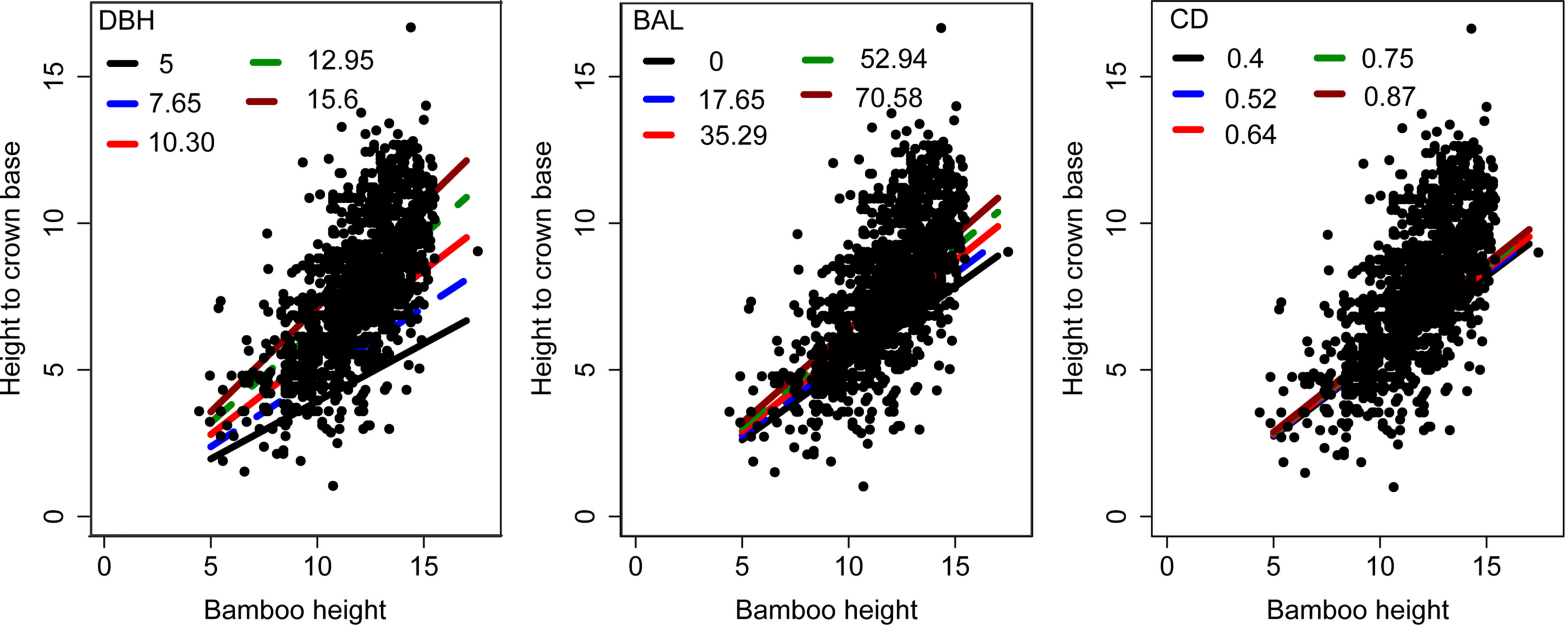
Base model (Eq. 11) simulation curves overlaid on scattered plot data (relationship between HCB and H).

The curves simulated using Eq. 11 (extended HCB model without random effects) passed almost through the middle of the data clouds (**Figure 5**), indicating that the model would be biologically reasonable, and the parameters easily explained.

### 3.3 NLME HCB model

**Figure 6:**
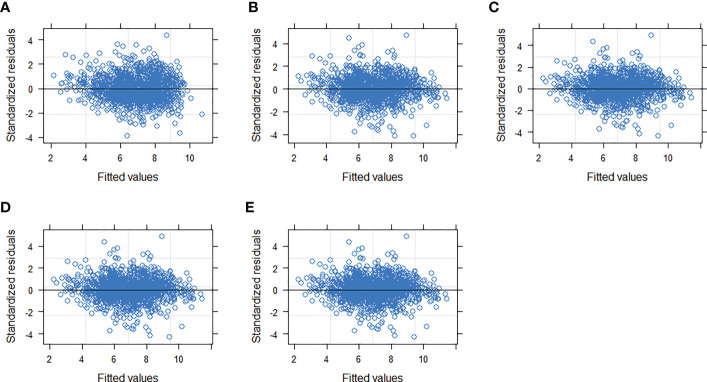
Residual distribution of the NLME HCB (Eq. 12) with and without using the variance functions. **(A)** OLS, **(B)** variance function not included, **(C–E)** different variance functions (Eqs. 6–8) included, respectively.


Random effects at both the block and sample plot levels were introduced into the extended model form (Eq. 11) and different parameter combinations were considered to determine the best combination of fixed-effect parameters and random effects. The NLME model using the combination of (Eq. 12) provided the smallest AIC (AIC = 4946) and maximum LL (LL= -2465), with the best-fit statistics (R^2^ = 0.6350, RMSE = 1.1285, TRE = 2.5742). Among the three evaluated variance functions (Eqs. 6–8), the exponential function (Eq. (6)) provided the smallest heteroscedasticity (**Table 4**; **Figure 6**).

**Table 4:** AIC and LL of different variance functions applied to NLME HCB model (AIC, Akaike Information Criteria; LL, loglikelihood).

Variance function	AIC	LL
None	4945.80	-2464.90
Eq. 6	4944.60	-2463.30
Eq. 7	4945.53	-2463.76
Eq. 8	4945.53	-2463.76

(12) $$HCB_{ijk}=\frac{H_{ijk}}{\left[1+e^{\left(\beta_{1}+\mu_{1i}+(\beta_{2}+\mu_{2i}+\mu_{2ij})DBH_{ijk}+\beta_{3}BAL_{ijk}+\beta_{4}CD_{ij}\right)}\right]}+\xi_{ijk}$$

All the parameters of the OLS HCB model (Eq. 11) and NLME HCB model (Eq. 12) were statistically significant (*p< 0.05*). The first model (Eq. 11), with estimated parameter values, is provided below (Eq. 13).


(13) $$HCB_{ijk}=\frac{H_{ijk}}{\left[1+e^{\left(1.3922-0.1273DBH_{ijk}-0.0068BAL_{ijk}-0.2494CD_{ij}\right)}\right]}+\xi_{ijk}$$

where $\xi_{ijk}\sim N(0,1.2920)$ , and similarly estimated NLME HCB model is,


(14) $$HCB_{ijk}=\frac{H_{ijk}}{\left[1+e^{\left(0.8407+\mu_{1i}+(-0.07922+\mu_{2i}+\mu_{2ij})DBH_{ijk}-0.0020BAL_{ijk}-0.3440\beta_{4}CD_{ij}\right)}\right]}+\xi_{ijk}$$

$$\text{where}\mu_{i}=\left[\begin{array}{c} \mu_{1i}\\ \mu_{2i} \end{array}\right]\sim N\left\{\left[\begin{array}{c} 0\\ 0 \end{array}\right],{\hat{\Psi }}_{1}=\left(\begin{array}{cc} 1.17e-02 & -0.529\\ -0.529 & 5.84e-05 \end{array}\right)\right\}$$

$$\mu_{2ij}∼N(0,2.13e-04)$$

$$\xi_{ijk}∼N(0,R_{i}=1.3017G_{i}^{0.5}\Gamma_{i}G_{i}^{0.5})$$

$${\hat{G}}_{ij}=diag[2.0048\exp(-0.0179H_{ij1}),\ldots ,2.0048\exp(-0.0179H_{ijk})\text{];}$$

$$\Gamma_{i}=I_{i}$$

### 3.4 Response calibration/model prediction

**Figure 7:**
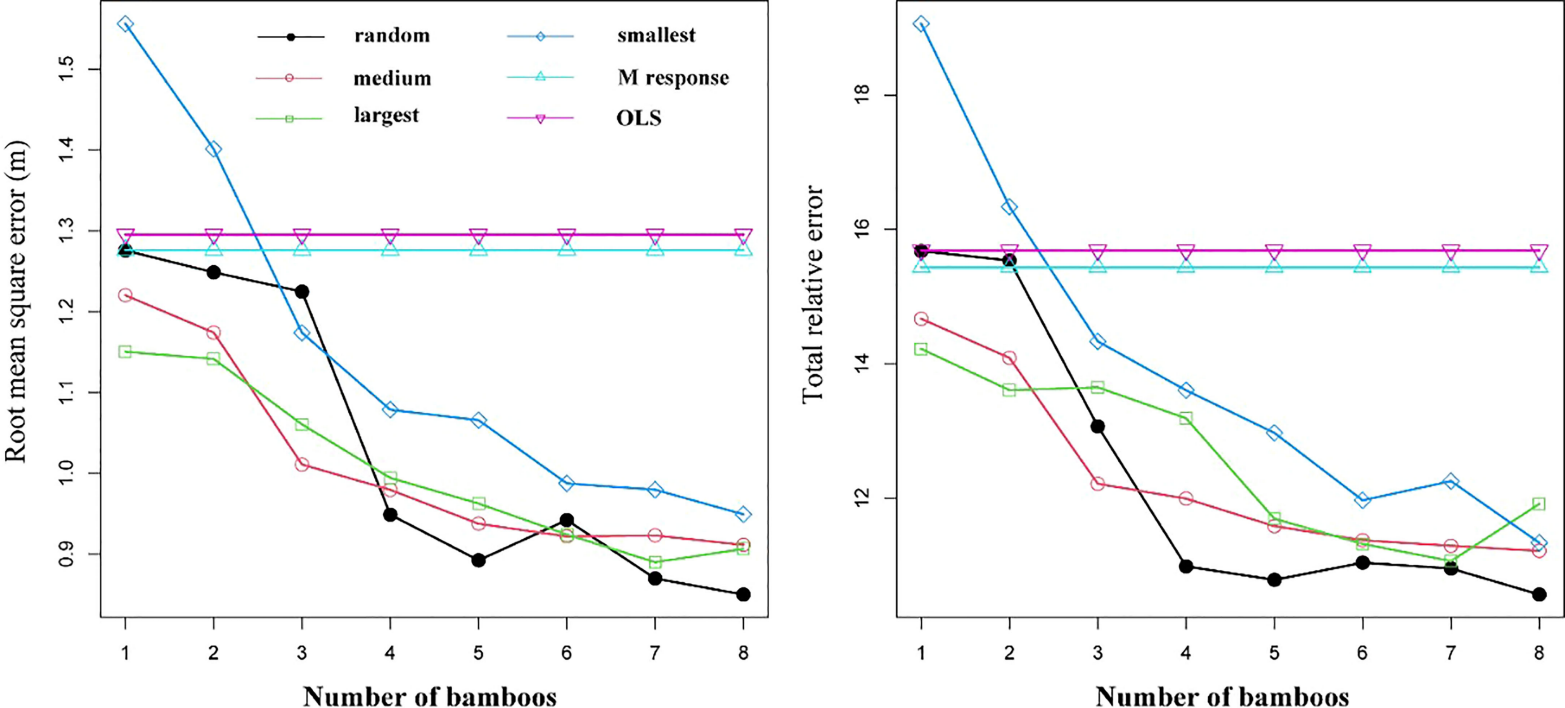
Root mean squared error (RMSE) and total relative error (TRE) for the ordinary least square (OLS) model (Eq. 11), mean response of NLME model (M response) (Eq. 12), and NLME model (Eq. 12) calibrated using four sampling strategies.

The results of using different sampling strategies to estimate random effects are shown in **Figure 7**. The different sampling strategies showed almost identical trends in prediction accuracy. With an increase in the number of samples, prediction accuracy incrementally improved. The RMSE and TRE of three of the sampling strategies were smaller than those of the M response and OLS models; the “smallest DBH” strategy was the exception (Eq. 11). When four randomly selected bamboo plants per sample plot were used, the model produced the maximum reduction rates of both RMSE and TRE, which were 22.53% and 16.54%, respectively. With the inclusion of additional samples, the prediction accuracy of the model incrementally improved, and while the cost and time for measurement also substantially increased. Thus, it would be reasonable to use four randomly-selected bamboo plants per sample plot for calibration of the NLME HCB model (Eq. 12).

### 3.5 Model evaluation

We used the same evaluation statistics (Eqs. 1–4) to compare the prediction performance of the OLS HCB model (Eq. 10) (RMSE = 1.2905; TRE=15.6863), NLME HCB model (Eq11) (RMSE = 1.1285; TRE=2.5742), and M response (RMSE = 1.2760; TRE=15.3380) model. Relative to the OLS and the M response models, the prediction statistics of the NLME HCB model were substantially improved. When using 1–8 randomly-selected bamboo plants to estimate the random effects at the block and sample plot level, the RMSE decreased from 0.04% to 33.4% relative to the M response of Eq. 12. This indicated that the random effects at the block and sample plot levels had a large impact on the change in HCB and their inclusion was justified.

## 4 Discussion

With growing concern regarding the increase in atmospheric CO_2_ concentration, researchers are increasingly paying attention to bamboo as a resource for reducing atmospheric CO_2_ because of its substantial advantages over woody plants, such as faster growth and carbon sequestration rates, high production capacity, and multiple uses (Zhang et al., 2014; Yen, 2015; Yen, 2016). Most studies emphasize the economic value of bamboo but pay little attention to its physiological growth characteristics, including HCB and CR dynamics, the quantification of which provides support for carbon stock estimation and global warming mitigation (Song et al., 2013; Yen, 2016).

In this study, among the six candidate models that were chosen from previous HCB modeling studies (Fu et al., 2017; Sharma et al., 2017; Yang et al., 2020; Zhou et al., 2022), the logistic model showed the best fitting effect (**Table 3**), which is consistent with HCB modeling studies on arboreal species (Fu et al., 2017; Pan et al., 2020; Yang et al., 2020). Moreover, the simulation curve of the model overlays the data, which is biologically reasonable, and the parameters are solved and easily explained (**Figure 5**). The two-level NLME HCB model developed with four predictor variables (H, DBH, BAL, and CD) was able to describe a large proportion of HCB variations, even though the data pattern was largely scattered (**Figure 3**). Some studies have used DBH and H as predictor variables in an HCB model (Rijal et al., 2012; Fu et al., 2017). Because DBH is the most important and accurate variable in forestry inventory, it has been widely used in forest modeling, including HCB modeling (Rijal et al., 2012; Fu et al., 2017; Zhou et al., 2021a). H reflects the vitality and competition of the plant. BAL reflects competition among individual bamboo plants in the sample plot and is widely used in various forest models (Zhang et al., 2017; Yang et al., 2020; Zhou et al., 2021a; Zhou et al., 2022). Our study also found a significant positive relationship between CD and HCB (R^2^ = 0.5226, RMSE = 1.2905, TRE = 3.3892) (**Figure 4**); however, the inclusion of CD was not attempted in previous HCB modeling studies, making our study novel. The inclusion of CD is necessary because it reflects the site quality and vitality of bamboo forests, with a larger CD meaning a denser bamboo crown, stronger bamboo vitality, and better site quality. The positive relationship between CD and HCB means that the larger the CD, the more bamboo in the forest, and the darker the environment under the bamboo forest, which is not suitable for photosynthesis, transpiration, and other physiological activities (Lawlor, 2009). Better growth and survival of bamboo would increase HCB, which improves adaptation to the environment.

Many researchers have found that stand density significantly contributes to HCB variation (Toney and Reeves, 2009; Russell and Weiskittel, 2011; Popoola and Adesoye, 2012). Stand density also reflects the competition among individual bamboo plants within a stand. The HCB of moso bamboo with the same DBH was larger in more-crowded bamboo forests. However, our study showed no significant impact of stand density on HCB. This may be due to the competition between individual bamboo plants, as reflected by BAL and CD.

We also evaluated other variables; however, their impact on the HCB model was minor. Although adding an increasing number of variables might slightly improve the accuracy of the model, introducing too many variables certainly leads to model non-convergence and biased parameter estimation caused by excessive parameterization (Fu et al., 2013; Fu et al., 2017). Including too many predictor variables in the HCB model raises inventory cost and takes longer.

Relative spacing may influence tree height and crown base relationships (Saud et al., 2016; Pan et al., 2020). However, this did not apply to our study because the bamboo stand density did not change much during a given period. While annual bamboo shoots may lead to a change in stand density, the bamboo in this study was cut down 4–6 years after it was planted, minimizing long-term stand density changes.

Our model introduced both block- and plot-level random effects, which largely reduced unexplained variance and improved the fitting accuracy of the model relative to the OLS model (**Figure 6**). The addition of random effects added to DBH produced the best results, which could be attributed to the DBH difference reflected in the data and expressed by the random effect.

Calibrating the mixed-effects model using small samples is important for applying this model in forestry. A small number of samples per stand could be used to determine the impact variables that significantly affect HCB (Liu and Cela, 2008). Calibration using various sample sizes has been evaluated and discussed in most studies (Calama and Montero, 2004; Yang et al., 2009; Campo et al., 2010; Fu et al., 2017; Ye et al., 2019; Yang et al., 2020; Zhou et al., 2021a). The prediction accuracy of the model can be significantly improved by increasing the number of samples (**Figure 7**). In our study, the use of four randomly selected bamboo plants resulted in the highest reduction rates of RMSE and TRE. Although increasing the number of samples led to improved prediction accuracy, it also increased inventory costs and lead time. Thus, considering these as limiting factors of the inventory, four randomly selected bamboo plants per sample plot are optimal for calibrating the mixed-effects model, as this provides a desirable compromise between measurement cost, model use efficiency, and prediction accuracy (Yang et al., 2009; Fu et al., 2017; Ye et al., 2019; Yang et al., 2020; Zhou et al., 2021a).

As pointed out in Section 1, the study of bamboo HCB is of great practical significance for research on bamboo forest photosynthesis, transpiration, and utilization. The proposed model (Eq. 12) predicts the HCB of bamboo forests under similar site conditions (stand density of 1000–4000 stem/ha, the slope of 0–15). It can provide a reliable basis for bamboo forest management, such as describing changes in the bamboo canopy, bamboo forest fire potential, and carbon storage (Ancelin et al., 2004; Kuprevicius et al., 2014; Yang, 2017).

Some studies have pointed out that there may be errors in HCB measurements (caused by the crew, equipment failure, etc.) (Omule, 1980; Fuller, 1987; Rencher and Schaalje, 2008; Gertner, 2011). In building the model, it was assumed that the response variable is random and error-free, and when any error is associated with this and the predictor variables, the resulting model may be significantly biased. However, we did not consider these potential sources of error in our study. If any variable in Eq. 11 contains significant errors, a new modeling method, such as error-in-variable modeling, must be developed to address this problem. Studies have shown that altitude and climate factors may also have a significant impact on HCB (Yang, 2017; Zhou et al., 2021a), which needs to be considered in future studies.

## 5 Conclusion

In this study, the NLME HCB model containing three individual bamboo variables (H, DBH, and total BAL or CD) was developed by introducing random effects at the block and sample plot levels. The NLME HCB model described the majority of HCB variations. The HCB increased with increasing DBH, CD, and BAL, with DBH having the greatest impact, followed by height and CD. There was a significant impact of the block- and sample plot-level random effects on HCB, and consequently, the NLME HCB model was significantly improved relative to the basic and expanded models without random effects (the fixed-effects model or M response). The NLME HCB model used in this study can be applied after calibration with random effects estimated using at least four randomly selected individual bamboo plants per sample plot and can accurately predict the HCB of bamboo forests under identical site conditions. The model will provide a reliable basis for bamboo forest management, such as describing changes in the bamboo canopy, bamboo forest fire potential, and carbon storage.

## Data availability statement

The original contributions presented in the study are included in the article/supplementary material. Further inquiries can be directed to the corresponding author.

## Author contributions

XiZ, XuZ, FG, GL, and SF designed this study and improved the English language and grammatical editing. XiZ wrote the first draft of manuscript, and performed the data analysis. XiZ, XuZ and YZ did the field works. XiZ and RS gave guidance and methodological advice. GL provided fund and funding support. All authors contributed to the article and approved the submitted version.
